# Whales and Bacteria

**Published:** 1891-03

**Authors:** 


### Whales and Bacteria.

It has been discovered that human blood has a germicidal action, and medical authorities are trying to discover who was the first to notice this action. Apropos to this we now also learn that for more than five hundred years the Norwegian fisherfolk have used pathogenic bacteria in catching whales, the animals are inoculated with an infectious disease and only after they are weakened as a result of the disease are they killed. As the poisoned arrows are pulled out of the whale, bacilli cling to them, and thus render them effective as "death arrows" when further used.

What next in the field of bacteriology? W.

To detect fecal matter in drinking water Griess recommends a feebly alkaline solution of para-diazo-benzol-sulphuric acid, which, with water contaminated as indicated, will produce a yellow discoloration within five minutes. Try it on the old well next summer.

WANTED.—The May number of the INDEPENDENT PRACTITIONER of 1881. Any one having this number will confer a favor by sending it to J. Taft, Cincinnati, O., to complete a file for a public library.

TINCTURE of iodine to moisten pumice stone is a good way with wooden points to remove the green and brown stain to be found on the teeth of the young. W.

KEROSENE oil will instantly remove the dirt from an Arkansas st)ne caused by sharpening instruments. Apply with a cloth.

To clean the hands from laboratory dirt and oil we have found Pyle's Pearline to be very efficient.

DENTAL engine hand-pieces should often be taken apart and cleaned with a lubricant that will not gum. Dr. Sillito uses filtrine, an excellent oil, and by fastening a small platina tube into a glass dropper at its point, the dropper is always charged for use. Use glass dropper which serves as a stopper for the bottle. The platina tube should be quite small to enable it to be placed into the parts of the hand-piece. W.

